# Enhanced strength and durability of long fiber type carbon fiber reinforced plastic rods over titanium alloy rods for spinal fixation

**DOI:** 10.1016/j.xnsj.2025.100608

**Published:** 2025-03-27

**Authors:** Kohei Morita, Hiroki Ohashi, Katsuhiro Oikawa, Satoshi Tani, Kostadin Karagiozov, Yuichi Murayama

**Affiliations:** a The Department of Neurosurgery, The Jikei University School of Medicine, Tokyo, Japan; b GLOBERIDE, Inc., Higashikurume, Japan; c The Department of Neurosurgery, Shin-Yurigaoka General Hospital, Kawasaki, Japan

**Keywords:** Carbon fiber-reinforced plastic, Durability, Internal fixators, Long fiber type, Orthopedic fixation devices, Polyaryletherketone, Safety, Spinal fusion, Spine, Titanium alloy rods

## Abstract

**Background:**

Carbon fiber-reinforced plastic (CFRP) has been used in many medical devices, including rods for posterior spinal fixation devices, owing to its superior radiolucency and durability compared with titanium alloys. However, the strength of the existing short fiber type CFRP does not surpass that of metals. Recently, the use of innovative technologies for manufacturing long fiber type CFRP has dramatically improved their strength. In this study, we developed a rod for posterior spinal fixation using long fiber type CFRP technology and evaluated its strength, durability, and radiolucency against rods made of titanium alloy, cobalt chrome, short fiber CFRP, and polyaryletherketone (PEEK).

**Methods:**

Comparison tests were conducted on the strength, durability, and image evaluation of long fiber type CFRP rods and titanium alloy rods. In addition, a series of tests required for regulatory approval and clinical use were conducted.

**Results:**

The long fiber type CFRP rod showed 120% of the strength of the titanium alloy rod, 102% of that of the cobalt chrome rod, and approximately 20 times the strength of the short fiber type CFRP and PEEK, demonstrating durability that remarkably exceeded that of the titanium alloy. Moreover, artifacts in the radiographic images were smaller than those observed with titanium alloy. Biosafety and magnetic resonance safety tests also yielded satisfactory results, supporting approval for clinical use.

**Conclusion:**

This study introduces a new type of long fiber type CFRP rod for spinal surgery that is stronger, lasts longer, and causes fewer imaging problems than current titanium rods, and may prevent complications such as rod breakage. The long fiber type CFRP rod may improve the safety and outcomes of spinal surgeries.

## Graphical abstract

**Figure:**
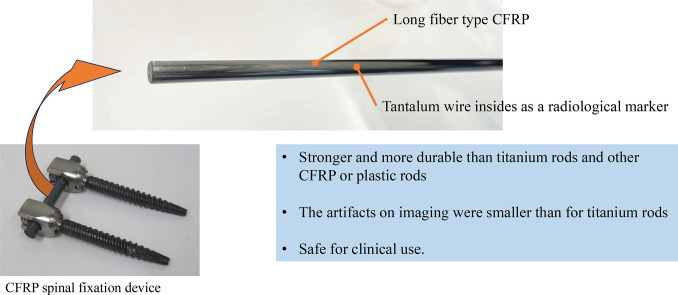

## Background

The global aging population is associated with increasing incidence of neurogenerative disease of the spine. Spinal degenerative diseases are progressive, and surgical intervention is considered in cases that are resistant to conservative treatment. Among surgical therapies, posterior fixation devices such as screws and rods are widely used. Although several fixation methods have been developed, the screw-and-rod system remains the most important construct, providing strong fixation.

In particular, the rod is an important part that determines fixation performance. Accordingly, many rods are made of titanium alloys owing to their strength, expressed as the value until failure under load; biocompatibility; and cost-efficiency [1]. Cobalt-chromium alloys are also used when stronger fixation is required [2].

However, metal rods are often broken by metal fatigue, compromising stability and resulting in strong artifacts in the surrounding area, as revealed in computed tomography or magnetic resonance imaging, making postoperative image evaluation difficult. Several ideas for solving these problems have been proposed, including using devices made of resins, such as polyaryletherketone (PEEK) and carbon fiber-reinforced plastics (CFRP) [[3], [4], [5], [6]]. However, rods made from PEEK or CFRP have not shown strength superior to that of metal rods used in fixation surgery. Consequently, they have not yet been used to replace metal rods [5,6].

By devising the molding method, length, and arrangement direction of the carbon fibers, CFRP is expected to have improved strength. The strength of CFRP depends on the length of the carbon fibers; the longer the fibers arranged in continuity, the greater the strength. However, considering the shape and size of the rods for spinal fixation devices, controlling the arrangement direction of the carbon fibers in CFRP molding is difficult, hindering the use of long carbon fibers; thus, molding with short fibers is inevitable (called the short fiber type).

With the development of fishing rods and CFRP materials for industrial use, technology has achieved a significant performance improvement over short fiber type CFRP by better controlling the pressure, temperature, and fiber orientation during molding. The CFRP fabricated in this manner is called the “long fiber type.” We applied this technology to fabricate high-strength, long fiber CFRP rods for posterior spinal fixation.

In this study, we compared the long fiber type CFRP rod made with this new technology against existing titanium alloy and cobalt-chromium rods to assess its performance and superiority. In posterior fixation, screw pullout and rod breakage are well-known complications related to reduced durability resulting from metal fatigue [7,8]. We expect that improving the performance of the rod will prevent these complications.

## Methods

### Performance evaluation methods

The performance of the rod must be evaluated for its strength and rigidity, which is defined as the degree to which a material deforms when a load is applied. High rigidity indicates that the material is stiff and does not bend easily. In posterior spinal fixation, rods that are highly rigid and difficult to bend are used to firmly fix the joint; however, materials with low rigidity such as PEEK bend and cannot be used in fixation surgery [9]. Therefore, when evaluating mechanical performance, simultaneous evaluation of strength and rigidity is necessary for determining whether the material is suitable for the posterior fixation procedure. In addition, evaluating the strength and durability (i.e. how long a material can withstand repeated loads of the same strength before breaking, with higher durability indicating a longer lifespan) of the permanently implanted rod is extremely important. Furthermore, the presence or absence of artifacts in the imaging findings is important in postoperative follow-up. Consequently, we planned a set of experiments to evaluate the strength, rigidity, durability, and interference (which occurs during radiological imaging examinations resulting with artifacts) during imaging evaluation of the long fiber CFRP rods.

### Manufacturing of long fiber CFRP rods

A rod with a diameter of 5.5 mm was produced, matching the shape and size of existing titanium alloy rods. For this study, the CFRP materials were produced with normal long carbon fiber and PEEK. Similar to general industrial and fishing rod manufacturing technology, the carbon fibers were aligned in a single direction during molding. The pressure applied into the mold with the material was adjusted to be high, and the temperature was set to the optimal value for the proportion of resin components.

### Strength and rigidity

**Fig. 1:**
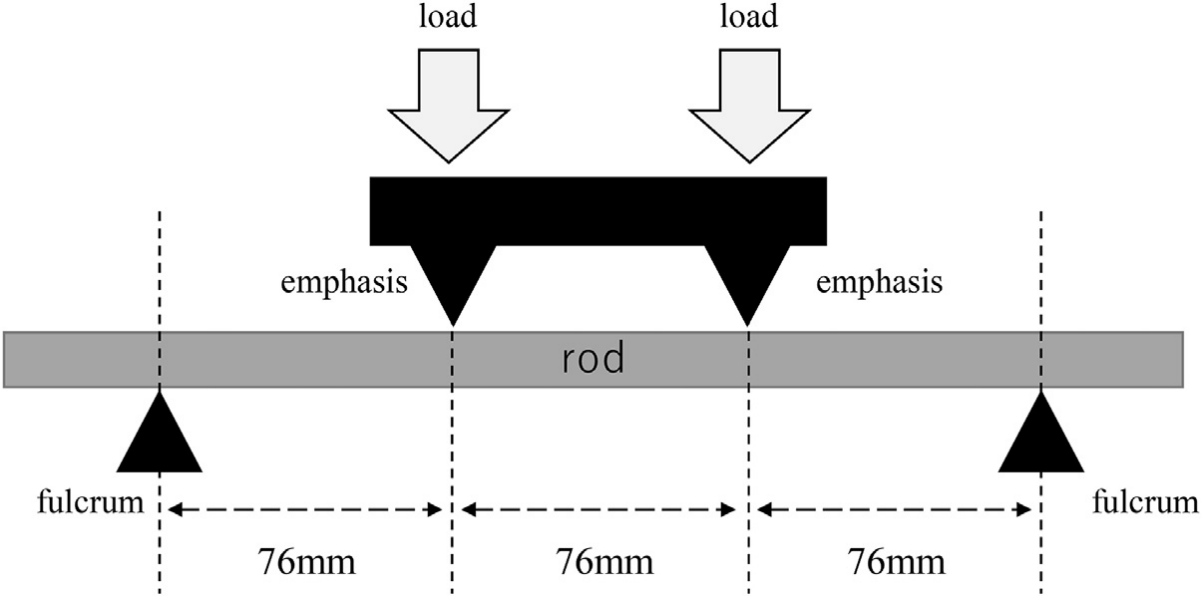
Load tests were conducted based on the American Society for Testing and Materials (ASTM) F2193. The strength of the rods was evaluated using 4-point bending at 76 mm intervals.

A 30-cm long rod was supported at 4 points, a load was applied to the center between the 2 points, and the bending force until breakage (maximum bending load) and rigidity (resistance to bending) under load were measured and evaluated (Fig. 1). The tests were conducted according to the international standards of the American Society for Testing and Materials (ASTM) F2193 “Standard Specifications and Test Methods for Components Used in the Surgical Fixation of the Spinal Skeletal System.” A jig was placed on the test device. The distance between the support and load rollers was 76 mm. The bending load was applied to achieve 10 mm/min deformation (sinking of the rod), as prescribed by ASTM regulator. The magnitude of the load was continuously measured, and the test was terminated when the specimen was significantly deformed.

Five rods (diameter 5.5 mm) made of long fiber type CFRP were used as test materials, and for comparison, 3 rods (diameter 5.5 mm) made of titanium alloy and cobalt-chromium, which are the most widely used rods globally (Solera™, Medtronic Sofamor Danek USA Inc., Memphis, USA), 3 rods of the same shape made of short fiber CFRP material using a conventional manufacturing method (diameter 5.5 mm, TECAPEEK CF30 black, Ensinger, Nufringen, Germany), and 5 or 3 rods of the same shape made of general PEEK material (diameter 5.5 mm, TECAPEEK, Ensinger, Nufringen, Germany) were used. For some materials that have already been used in clinical or industrial applications and have small variations and variances in performance can be used reduced number of samples (N), there the N number was set to 3. The strength tester ElectroPuls E3000 (Instron, Norwood, MA, USA) was used for the test.

### Durability

The tests were conducted according to ASTM F2193 international standards. The rod was supported at 4 points, and a load was applied to the 2 center points to evaluate the degree of fatigue of the rod due to repeated loads. The specimen was prepared in the same manner as that for the strength and stiffness measurements and was attached to the test equipment. A repeated bending load was applied at a target test frequency of 5 Hz. The test load was determined from the results of the strength and stiffness tests. The test was terminated when the specimen was significantly deformed or reached 2.5 million repetitions without breakage. Four long fiber type CFRP rods (diameter 5.5 mm) were used as test materials, and 4 titanium alloy rods (diameter 5.5 mm) (Solera™, Medtronic Sofamor Danek USA Inc.) were used for comparison. The test equipment was identical to that used for the strength and stiffness measurements.

### Artifacts during image evaluation

Image interference was evaluated using computed tomography (CT) and magnetic resonance imaging (MRI) scans. For CT examination, the rod was implanted in a swine, and images were obtained using cone-beam CT scanning. One long fiber type CFRP rod (diameter 5.5 mm) was used as the test material, and one titanium alloy rod (diameter 5.5 mm) (Solera™, Medtronic Sofamor Danek USA Inc.) was used as the comparison object. The Infinix Celeve-i system (Canon Medical Systems, Ohtawara, Japan) was used for imaging.

For MRI examination, ASTM F2119 “Standard Test Method for Evaluation of MR Image Artifacts from Passive Implants.” A phantom was prepared using 1.32 g/L NaCl and 10 g/L partial sodium salt of polyacrylic acid (PAA) (436364 CAS no. 76774-25-9, poly [acrylic acid] partial sodium salt, <1000 μm particle size [99%]). One long fiber type CFRP rod (diameter 5.5 mm) was used as the test material, and one titanium alloy rod (diameter 5.5 mm) (Solera™, Medtronic Sofamor Danek USA Inc.) was used as the control. These materials were positioned within the phantom, and images were captured using spin echo and gradient echo sequences. The artifacts were quantified by measuring the artifact distance (mm) from the boundary of the test materials to the outer edge of the artifact. Imaging was performed using a 3T MRI scanner (MAGNETOM Vida system, Siemens Healthineers AG, Erlangen, Germany).

Stata 18 (StataCorp, College Station, TX, USA) was used for statistical analyses to evaluate the results of this study. Differences between the features and effects of the 2 types of rods were analyzed using Student's t-test. A p-value < .05 was considered significant.

### Biosafety and other tests

For medical devices implanted in the body for long periods, it is essential to conduct comprehensive tests to confirm safety for approval and clinical use. We conducted biological safety tests and MR safety tests necessary for safe MRI imaging to compare the long fiber type CFRP rods (diameter 5.5 mm) with the titanium alloy rods (diameter 5.5 mm) (Solera™, Medtronic Sofamor Danek USA Inc.). For assessing biological safety, tests for cytotoxicity, skin sensitization, intracutaneous reactivity, pyrogenicity, acute systemic toxicity, reverse mutations, chromosomal aberrations, and implantation were performed based on ISO 10993. The implantation test was conducted over 24 weeks to confirm long-term safety, with evaluations of the internal organs, skin, and peri-implant tissues, according to standard ISO 10993 test. In this series of evaluations, all animal-related testing was conducted in accordance with ISO 10993-2:2022 Biological Evaluation of Medical Devices-Part 2: Animal welfare requirements.

In addition to the artifact evaluation test mentioned above, MR safety tests were conducted in accordance with ASTM F2052 “Standard Test Method For Measurement Of Magnetically Induced Displacement Force On Medical Devices In The Magnetic Resonance Environment,” F2213 “Standard Test Method for Measurement of Magnetically Induced Torque on Medical Devices in the Magnetic Resonance Environment,” and F2182 “Standard Test Method for Measurement of Radio Frequency Induced Heating On or Near Passive Implants During Magnetic Resonance Imaging.” These tests measure and evaluate the torque and deflection forces applied to the device in a magnetic field, as well as the heat generation. The MRI equipment used was a 3T MRI, as also used in the artifact measurement test.

## Results

### Strength and rigidity

**Fig. 2:**
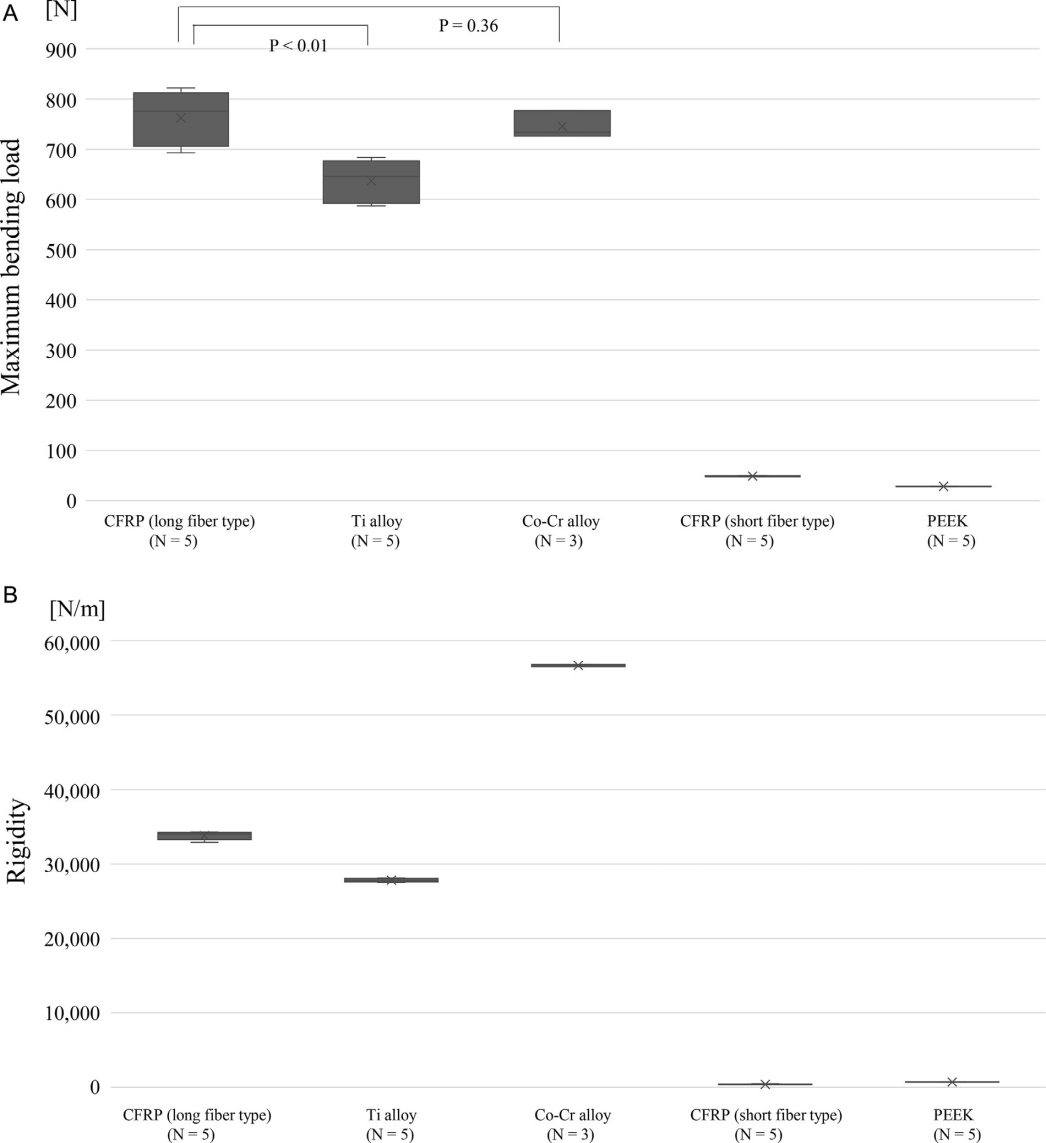
(A) Maximum bending load for each material. (B) Rigidity for each material.

At the maximum bending load, the titanium alloy rod broke at 637.0 N ± 43.0 N (mean ± standard deviation), the cobalt-chromium rod at 745.7 N ± 27.4 N, while the long fiber type CFRP rod had a strength of 762.8 N ± 54.8 N (Fig. 2A). For comparison, the strength of the PEEK rod was 28.4 N ± 0.5 N, while that of the short fiber CFRP rod was 48.8 N ± 0.8 N. The rigidity was 33.8 kN/m ± 0.6 kN/m for the long fiber type CFRP rods, 27.8 kN/m ± 0.2 kN/m for the titanium alloy rods, 56.7 kN/m ± 0.2 kN/m for the cobalt-chromium rods, 0.4 kN/m ± 0.1 kN/m for the short fiber CFRP rods, and 0.7 kN/m ± 0.0 kN/m for the PEEK rods (Fig. 2B). Overall, the stiffness of the long fiber CFRP rods was not significantly different from that of the titanium and cobalt-chromium rods; however, the maximum loading values of the long fiber CFRP rods were significantly higher than that of the titanium rods (p < .01) and tended to exceed that of the cobalt-chromium rods.

### Durability

**Fig. 3:**
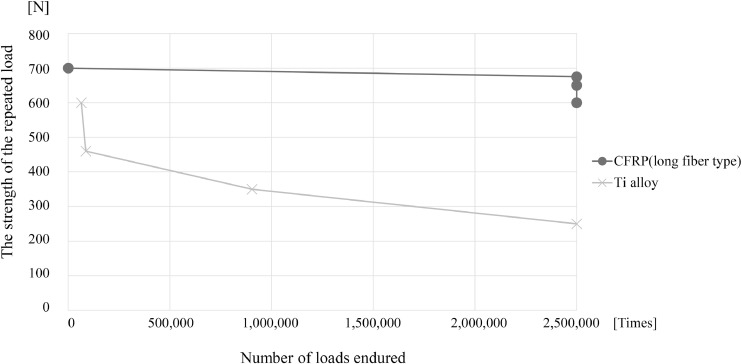
Comparison of durability between long fiber type CFRP rod and titanium alloy rod.

In the fatigue test of the rod alone, the titanium alloy rod withstood 2.5 million cycles under a load of 250 N and broke at 903 515 cycles under 350 N, at 86 796 cycles under 460 N, and at 64 053 cycles under 600 N, whereas the long fiber type CFRP rod subjected to 2.5 million cycles of loads of 600, 650, and 675 N did not break. This process ceased after 242 cycles at 700 N. The long fiber CFRP rod was remarkably more resistant to fatigue than the titanium alloy rod (Fig. 3).

### Artifacts during image evaluation

**Fig. 4:**
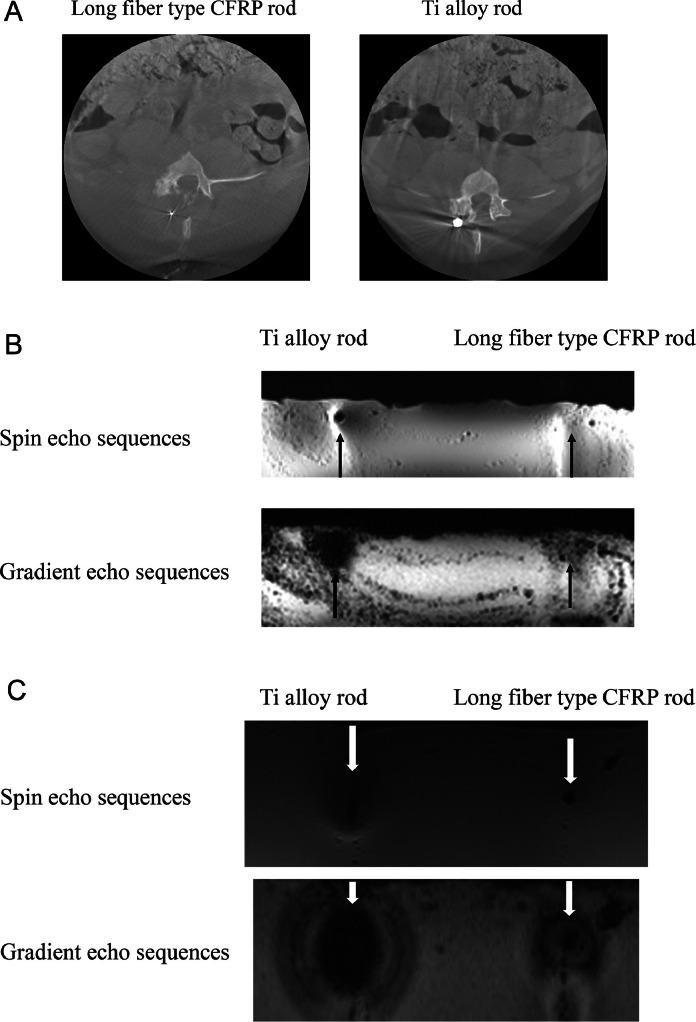
(A) Cone-beam computed tomography images. A tantalum wire was embedded centrally in the long fiber type CFRP rod to confirm its position under fluoroscopy; the high-density area is the resulting artifact. (B) Magnetic resonance imaging (3T). Long fiber type CFRP rod artifacts are smaller than those of titanium alloy rods. (C) Magnetic resonance imaging (1.5T).

The long fiber type CFRP rod showed a clear reduction in artifacts compared with the titanium alloy rod on CT (Fig. 4A) and MRI images (Fig. 4B, C). No artifacts were observed in the parts using CFRP other than the tantalum alloy implanted as a marker for position and damage evaluation during and after surgery.

### Biosafety and other tests

The long fiber CFRP rod demonstrated biological safety equivalent to that of the titanium alloy in biological safety tests, with no observed toxicity. MR safety tests indicated that torque force, deflection force, and heat generation were comparable to or less than that of the titanium alloy rod (Table 1).

**Table 1:** Biological safety tests and MR safety tests for approval and clinical use.

Tests		Results	
		Long fiber CFRP rod	Ti alloy rod
Biosafety tests (ISO 10993)	Cytotoxicity	No toxicity	No toxicity
	Skin sensitization	No toxicity	No toxicity
	Intracutaneous reactivity	No toxicity	No toxicity
	Pyrogen	No toxicity	No toxicity
	Acute systemic toxicity	No toxicity	No toxicity
	Reverse mutation	No toxicity	No toxicity
	Chromosomal aberration	No toxicity	No toxicity
	Implantation (24 wk)	No toxicity	No toxicity
MR safety tests	ASTM F2052 “Standard test method for measurement of magnetically induced displacement force on medical devices in the magnetic resonance environment”	No displacement	No displacement
	ASTM F2213 “Standard test method for measurement of magnetically induced torque on medical devices in the magnetic resonance environment”	Extremely weak torque, less than Ti alloy rod	Extremely weak torque
	ASTM F2182 “Standard test method for measurement of radio frequency induced heating on or near passive implants during magnetic resonance imaging”	Extremely weak heating, less than Ti alloy rod	Extremely weak heating

MR, magnetic resonance; ASTM; The American Society for Testing and Materials.

## Discussion

### Advantages of long fiber CFRP rod application

In medicine, PEEK has been widely used for vertebral cages and other devices owing to its flexibility, high biocompatibility, and ease of processing [3]. CFRP has also been widely used for fracture plates and other devices because of its excellent flexibility, high biocompatibility, and higher strength compared with resins [10]. These resins and CFRP have also been used for rods. Although these PEEK and short fiber type CFRP rods are visible on radiographic images, none exhibited a strength equivalent to that of metals [[4], [5], [6],[11], [12], [13], [14], [15], [16], [17], [18], [19], [20], [21], [22], [23], [24], [25], [26], [27]]. As their strength and rigidity are significantly lower than those of metals, comparing them with metals as fixation devices has been difficult. Owing to this, their use in clinical practice never expanded toward replacing metal rods.

However, the long fiber type CFRP rods showed a stiffness equivalent to that of the titanium alloy and cobalt-chromium rods. Furthermore, the maximum loading values of the long fiber type CFRP rod was significantly higher, measuring at 120% of that of titanium alloy rods, 102% of that of cobalt-chromium rods, and approximately 20 times that of PEEK and existing short fiber type CFRP rods. It far surpassed the resin or short fiber CFRP rods used in the past and appeared to be sufficiently strong to surpass existing metal rods. Hence, the strength of the long fiber CFRP rods is sufficient for use in clinical practice.

In terms of durability, the long fiber type CFRP rod was significantly more resistant to fatigue than titanium rods. In addition to the similar properties of resin and short fiber type CFRP rods, [[4], [5], [6],^15^]. the long fiber type CFRP rod also exhibited durability and resistance to fatigue. This may be attributed to the vibration absorption properties of the CFRP, which can easily absorb fine vibrations and micro-movements compared with metals [28,29]. Considering the results of this study, the long fiber type CFRP rod surpassed metal rods in terms of both strength and durability. As rod breakage is a frequent adverse event, the application of long-fiber CFRP may significantly reduce postoperative complications.

In image evaluation, rods made of resin or short fiber type CFRP showed good results in terms of low image analysis interference compared with metal rods [[11], [12], [13], [14],^16^,^21^,^22^,[24], [25], [26], [27]]. The long fiber type CFRP rod also showed a similar low interference level, superior and suitable for practical purposes compared with metal rods. Metallic devices often make it difficult to assess bone union and visualize structures inside of the spinal canal after surgery, but with CFRP implants, these assessments can be conducted easily, contributing to improved safety in postoperative management.

### Clinical application

The long fiber type CFRP rod outperformed metal rods in terms of strength, durability, and artifacts, and presented potential clinical value. Neither biological nor MRI-based safety concerns were observed, suggesting the material may be suitable for regulatory approval and clinical use, once its clinical application advantages have been validated.

First, postoperative device breakage risk must be assessed. Rod breakage occurs frequently and delays vertebral fusion. Affected patients require reoperation, which is associated with physical, psychological, and socio-economic burden [30,31]. In most cases, rod breakage occurs after surgery rather than during surgery, likely due to repeated loading, suggesting the need for a material with improved durability. CFRP has vibration absorption properties, and unlike metals, it does not "fatigue." In this study, the durability of long fiber type CFRP rods was superior to that of metals. Consequently, it is expected that the use of the long fiber type CFRP rods in clinical practice could reduce the risk of complications from rod breakage, helping improve outcomes and optimize resource use.

Second, spine surgeons sometimes have difficulty judging the degree of bone union on follow-up scans due to metal artefacts on images of the structures located near the spinal canal. With a CFRP device, artefacts are less pronounced, helping improve follow-up assessments. Although existing short fiber type CFRP devices are superior in this respect, they cannot be used for patients with degenerative diseases due to their mechanical properties; long fiber type devices can be eventually used in such cases.

**Fig. 5:**
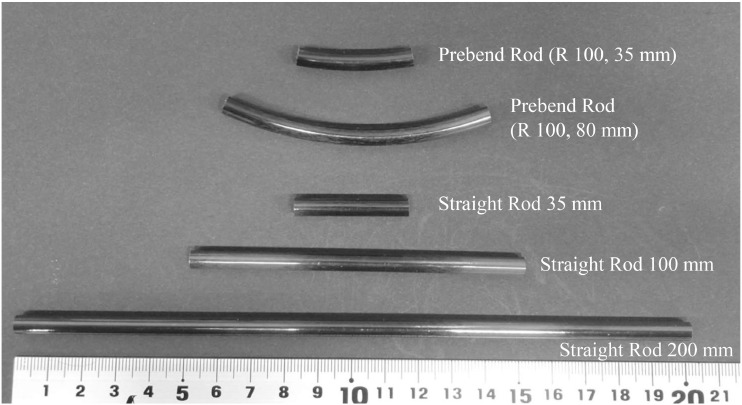
Partial lineup of long fiber CFRP rods under consideration for clinical use.

Finally, titanium alloy rods are available in a wide variety of shapes and can be bent or cut during surgery. The substrate of the long fiber type CFRP rod is made of thermoplastic resin, which means it could be heated with an appropriate device for the purpose and shaped during surgery. However, as is the case with metal rods, a CFRP rod has some limitations regarding the extent to which it can bended. To prevent the uncertainty of an increase in postoperative breakage risk due to improper use, we initially setup a large lineup of shapes and lengths, including preshaped types, which should be available at the time of clinical application (Fig. 5). In addition, we are currently refining and testing the method for bending during surgery.

Although it is theoretically possible to cut the rod during surgery, this approach increases the risk of damage to the fiber structure. Therefore, we are planning to offer initially a wide range of rod lengths and gradually adapt cutting methods.

Despite some still existing limitations to their use, long fiber type CFRP rods might be applied soon instead of titanium alloy rods in clinical settings, as we are in the process of application for regulatory approval as part of our future protocol.

## Conclusion

In summary, the long fiber CFRP rod with the new molding technology exhibited superior mechanical performance and reduced imaging artifacts compared with existing titanium alloy rods. Furthermore, it had acceptable biosafety and is therefore a viable alternative to current metal rods in spinal fixation. Additionally, the manufacturing cost is comparable to that of existing titanium alloys, suggesting economic benefits. This device can contribute to improving postoperative functional prognosis and stabilizing image follow-up and is expected to replace titanium alloy rods in clinical practice.

## Funding

This research was a project supported by the Japan Agency for Medical Research and Development, a public agency of the Japanese government, for the years 2019 to 2021 and 2023 to 2024 (ID: 24he1022012h0002).

## Declaration of competing interests

One or more of the authors declare financial or professional relationships on ICMJE-NASSJ disclosure forms.
